# Draft Genome Sequence of “*Candidatus* Liberibacter asiaticus” from a Citrus Tree in San Gabriel, California

**DOI:** 10.1128/genomeA.01508-15

**Published:** 2015-12-23

**Authors:** F. Wu, L. Kumagai, G. Liang, X. Deng, Z. Zheng, M. Keremane, J. Chen

**Affiliations:** a Department of Plant Pathology, Laboratory of Insect Ecology, South China Agricultural University, Guangzhou, Guangdong, China; b Plant Pest Diagnostic Center, California Department of Food and Agriculture, Sacramento, California, USA; c USDA-ARS, National Clonal Germplasm Repository for Citrus and Dates, Riverside, California, USA; d San Joaquín Valley Agricultural Sciences Center, Parlier, California, USA

## Abstract

The draft genome sequence of “*Candidatus* Liberibacter asiaticus” strain SGCA5 from an orange citrus tree in San Gabriel, California, is reported here. SGCA5 has a genome size of 1,201,445 bp, a G+C content of 36.4%, 1,152 predicted open reading frames (ORFs), and 42 RNA genes.

## GENOME ANNOUNCEMENT

“*Candidatus* Liberibacter asiaticus” is an unculturable phloem-limited alphaproteobacterium associated with citrus huanglongbing (HLB), a highly destructive disease in citrus production (1, 2). HLB was not known to be in the United States until 2005, when the first case was reported in the Florida (3). In California, “*Ca.* Liberibacter asiaticus” was first detected in a single citrus tree in Hacienda Heights of Los Angeles County in 2012 (4). The infected tree was quickly removed as part of the HLB eradication efforts. The whole genome of a Hacienda Heights strain (HHCA) has been reported (5). In 2015, the California Department of Food and Agriculture detected “*Ca.* Liberibacter asiaticus” in 10 more citrus trees in San Gabriel City, adjacent to Hacienda Heights. To better understand the California strains of “*Ca.* Liberibacter asiaticus,” this study sequenced the whole genome of a San Gabriel strain obtained from an orange tree displaying HLB symptoms.

DNA of “*Ca.* Liberibacter asiaticus” strain SGCA5 was extracted from petiole and leaf midrib tissue using the DNeasy plant minikit (Qiagen, Valencia, CA). The concentration of “*Ca.* Liberibacter asiaticus” was estimated by PCR (6), with a threshold cycle (*C_T_*) value of 20. The enrichment-enlargement procedure of Zheng et al. (7) was adapted for whole-genome sequencing. Briefly, bacterial DNA was enriched using the NEBNext microbiome DNA enrichment kit (New England BioLabs, Inc., Ipswich, MA) and enlarged with the illustra GenomiPhi version 2 DNA amplification kit (GE Healthcare, Inc., Waukesha, WI). The amplified DNA was sequenced with the Illumina MiSeq format (Illumina, San Diego, CA).

A total of 3.82 × 10^7^ reads with a mean of 251 bp per read were generated from MiSeq sequencing. Using our “*Ca.* Liberibacter asiaticus” genome strain YCPsy (accession no. LIIM00000000) (8) as a reference, a total of 61,528 reads with 15,443,528 bases were identified using standalone BLAST software (version 2.2.30; e-value, <10^-5^) (9). The “*Ca.* Liberibacter asiaticus” reads were collected using a Perl script. The referenced assembly generated 55 contigs ranging from 1,039 bp to 155,151 bp, with an average coverage of 12×, using Bowtie 2 (version 2.2.6) (10). Annotation was performed by the RAST server (http://rast.nmpdr.org) (11). The draft genome of “*Ca.* Liberibacter asiaticus” strain SGCA5 is composed of 1,201,445 bp, which is 97.39% of the YCPsy genome (1,233,647 bp), with a G+C content of 36.4%, 1,152 predicted open reading frames (ORFs), and 42 RNA-coding genes.

### Nucleotide sequence accession number.

The draft genome sequence has been deposited in DDBJ/EMBL/GenBank under the accession number LMTO00000000. The version described in this manuscript is the first version.
